# Exploring Demographic Representation and Reporting in Lung Cancer Clinical Trials with Canadian Sites from 2013 to 2023

**DOI:** 10.3390/curroncol31090413

**Published:** 2024-09-17

**Authors:** Sierra A. Land, Rajvi J. Wani, Naila Inam, Hilary J. G. Hewitt, Paulo Eduardo Muniz Covizzi, Tarah Sheculski Rivard

**Affiliations:** 1 Amgen Canada Inc., 6775 Financial Drive, Suite 300, Mississauga, ON L5N 0A4, Canada; sierraland4@gmail.com (S.A.L.); nailainam@gmail.com (N.I.); hhewitt@amgen.com (H.J.G.H.); pcovizzi@amgen.com (P.E.M.C.); trivard@amgen.com (T.S.R.); 2 Faculty of Medicine, University of Ottawa, Roger Guindon Hall, Ottawa, ON K1H 8M5, Canada

**Keywords:** representation, reporting, lung cancer, clinical trial diversity, Canadian population, minority populations

## Abstract

This review evaluates the reporting of demographic characteristics and the diversity of participants of phase III lung cancer clinical trials with Canadian research sites. A literature search was conducted using the ClinicalTrials.gov registry to identify clinical trials conducted between 1 January 2013, and 31 December 2023. The demographic reporting practices and the representation of sex/gender, racial, and ethnic groups were assessed. The location of Canadian research sites was also examined for trends in reporting and representation. Associated publications were reviewed for demographic data collection methods. Of the 25 clinical trials, 24 reported race and 18 also reported ethnicity. All clinical trials reported sex/gender, and the city and province of the participating Canadian sites. Most participants were White (66.1%), identified as not Hispanic or Latino (81.4%), and were male (57.8%). The provinces with the most clinical trial sites were Ontario (43.6%) and Quebec (34.2%). Lung cancer clinical trials lack adequate demographic reporting and representation of females, diverse patient groups, and geographical locations in Canada with high lung cancer incidence rates. Specifically, the Indigenous Peoples of Canada and Nunavut require better representation in lung cancer clinical trials conducted in Canada. These findings highlight the need to improve diversity and demographic representation in clinical research.

## 1. Introduction

Lung cancer is one of the most commonly diagnosed cancers with approximately 2.2 million new cases annually (11.7% of all sites) and it is the leading cause of cancer deaths with approximately 1.8 million annual deaths (18.0% of all sites) worldwide [1,2]. Of the two subtypes of lung cancer, non-small cell lung cancer (NSCLC) makes up most of the cases (80–90%) compared to small cell lung cancer (SCLC) [3]. In Canada, an estimated 31,000 new lung cancer cases and 20,600 lung cancer deaths were expected in 2023 [4,5]. Canadian lung cancer incidence rates vary based on sociodemographic and geographic factors such as sex/gender, racial and ethnic background, and location of residence. While historically higher among males, the gap has been narrowing and the current projected incidence rates for lung and bronchus cancer are around 60.1 per 100,000 person-years for males and 58.4 for females [4,5]. Among the various racial and ethnic groups in Canada, Indigenous Peoples (First Nations, Métis, and Inuit) have the highest lung cancer incidence rate (89.7 per 100,000 person-years), followed by White individuals (70.5 per 100 person-years) and people of East/Southeast Asian descent (44.3 per 100 person-years) [6]. Geographically, Nunavut is home to a large Indigenous population (85.8%) and has the highest lung cancer incidence rate (160.2 per 100,000 people), which may be partially due to the high smoking rate (62.1%) [7,8,9,10,11]. The Atlantic provinces, Quebec, and the Northwest Territories have similar lung cancer incidence rates ranging from 86.7 to 90.2 per 100,000 people [8,11].

Factors such as stress and social exclusion may prompt individuals to adopt negative coping mechanisms such as smoking and increase their risk of developing lung cancer [12]. These factors may disproportionately affect minority groups, including lesbian, gay, bisexual, transgender, queer and questioning, and two-spirit (LGBTQ2S+) individuals [13] as well as Indigenous communities [12]. Additionally, rural and remote areas are more likely to have higher lung cancer rates and more cases diagnosed at advanced lung cancer stages due to limited access to healthcare [14,15]. Indigenous Peoples of Canada are particularly affected, given that four in ten live in rural and remote communities [14].

Most lung cancer cases in Canada (around 70%, excluding Quebec where data on cancer stages is lacking) are diagnosed at stages III and IV when treatment options are limited and survival rates are low [16]. However, ongoing clinical trials offer hope for potential breakthroughs. Clinical trials produce novel cancer treatments such as targeted therapies to treat specific mutations and genomic profile-guided adjuvant therapies [17]. Since genomic alterations are linked with race and ethnicity, targeted therapies highlight the importance of reporting diverse factors and representation of diverse races and ethnicities in clinical trials [18]. While personalized medicine shows potential for improving health outcomes, it may worsen disparities in healthcare, especially for specific ethnic-cultural minorities, those with low socioeconomic status, and individuals lacking access to genetic testing and other healthcare services [17,19]. Both historically and currently, there are concerns regarding the underrepresentation of certain minority groups, such as Indigenous Peoples, LGBTQS2+, and females in clinical trials [20,21,22,23,24]. Most of the literature regarding genomic alterations in lung cancer is with Asian and White cohorts [18], and biorepositories used in genome-wide association studies are mostly populated with samples from individuals of European ancestry [18,25]. Since clinical trials offer access to the latest treatments and their results inform treatment decisions, it is important to understand if these clinical trials reflect the increasingly diverse Canadian population. The incidence rates for racial and ethnic groups in Canada are different compared to their regions of origin, highlighting the impact of social determinants of health, lifestyle, and early life exposures [6,26].

There have been increasing efforts to promote proportional representation in clinical trials and the reporting of demographic information, both on a global scale and within Canada. “The Sex and Gender Equity in Research (SAGER)” international guidelines provide a framework for sex and gender reporting [27], and the United States-based National Institutes of Health (NIH) and the Office of Management and Budget (OMB) have guidelines for race and ethnicity reporting [28,29]. In 2017, the NIH made it a requirement for race and ethnicity to be reported on the ClinicalTrials.gov registry [29,30]. In Canada, Health Canada launched a mandatory questionnaire in October 2022 as part of a multi-step approach to collect disaggregated data from regulatory submissions for new drugs [31]. The questionnaire reports on the populations included in the clinical trials in terms of sex, age, and race/ethnicity. This demonstrates an interest from regulatory authorities to mandate transparency around demographic data, which may in turn improve the representation of participants involved in the drug development process [31]. The survey has limitations as it does not inquire about gender, and only asks whether data are disaggregated by sex, age, or race/ethnicity without delving into how the demographic information is collected (e.g., the race/ethnicity categories used) [32]. Additionally, the Canadian Institute for Health Information (CIHI) [33] and the Canadian Medical Association Journal (CMAJ) [34] recently published standards and guidance for the reporting of race and ethnicity information in healthcare and health research, respectively.

The aim of this review is to evaluate how well the Canadian lung cancer population is represented in clinical trials and to provide context for Canadian healthcare providers interpreting the clinical trial results. The representation of demographic diversity and reporting of lung cancer clinical trials with Canadian sites were assessed.

## 2. Materials and Methods

### 2.1. Search Strategy and Data Extraction

**Figure 1:**
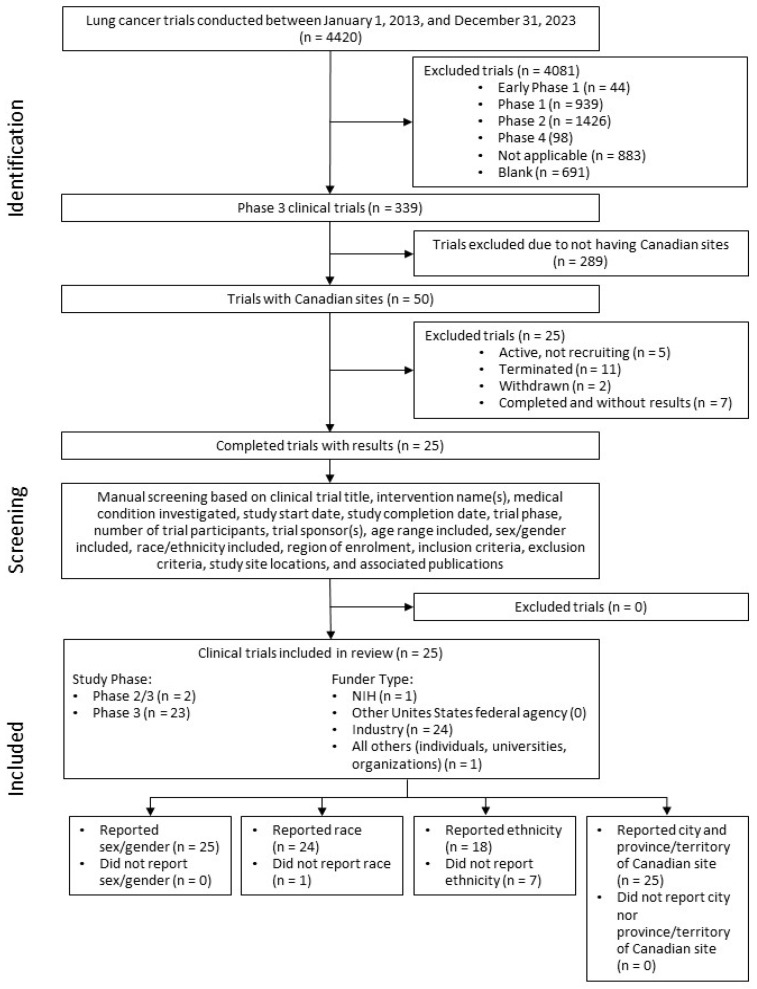
Flow chart of literature search.

A literature search was conducted using www.ClinicalTrials.gov (accessed on 1 March 2024), an online database of clinical research trials with information regarding their results, to identify clinical trials started between 1 January 2013, and 31 December 2023 (Figure 1). “Lung cancer” was the search term under the “Condition/disease” filter. “Canada” was the search term under the “Location” filter to ensure that only clinical trials with Canadian research sites were included. “Completed” clinical trials “with results” were additional filters applied to ensure trial results with complete participant demographics were included. “All” was chosen as the filter for “Sex” and “Phase 3” was the filter for “Study Phase”. The resulting clinical trials were then manually screened for clinical trial title, intervention name(s), medical condition investigated, trial start date, trial completion date, trial phase, number of trial participants, trial sponsor(s), age range included, sex/gender included, race and ethnicity included, region of enrolment, inclusion criteria, exclusion criteria, trial site locations, and associated publications. The location of Canadian clinical trial sites was assessed for trends in reporting and representation. The associated publications were reviewed to identify how sex/gender, race, and ethnicity information were collected.

### 2.2. Cohort Categories Used by ClinicalTrials.gov and Definitions

Reporting of participant sex and gender data on ClinicalTrials.gov was completed under Sex: female, male or Sex/Gender, which included categories for male, female, and undifferentiated. The definitions used by ClinicalTrials.gov for sex and gender are as follows: Sex refers to “a person’s classification as male or female based on biological distinctions”, whereas gender refers to “a person’s self-representation of gender identity” [35].

Race and ethnicity are both social constructs used to categorize people into groups [33,36]. Race focuses on perceived differences in physical traits, whereas ethnicity is based on community affiliation and sociodemographic characteristics (e.g., geographic origin, language, cultural traditions) [33,36].

Reporting of participant race data on ClinicalTrials.gov was completed under the following cohort categories: American Indian or Alaska Native, Asian, Black or African American, Native Hawaiian or other Pacific Islander, White, More than one race, Other, and Unknown or not reported. American Indian or Alaska native refers to “A person having origins in any of the original peoples of North and South America (including Central America), and who maintains tribal affiliation or community attachment” [30]. Asian refers to “A person having origins in any of the original peoples of the Far East, Southeast Asia, or the Indian subcontinent including, for example, Cambodia, China, India, Japan, Korea, Malaysia, Pakistan, the Philippine Islands, Thailand, and Vietnam”. Black or African American refers to “A person having origins in any of the black racial groups of Africa”. Native Hawaiian or other Pacific Islander refers to “A person having origins in any of the original peoples of Hawaii, Guam, Samoa, or other Pacific Islands”. White refers to “A person having origins in any of the original peoples of Europe, the Middle East, or North Africa” [30].

Reporting of participant ethnic data on ClinicalTrials.gov was completed under the following cohort categories: Hispanic or Latino, Not Hispanic or Latino, Unknown or not reported, Hispanic, Latino, or Spanish, and Not Hispanic, Latino, or Spanish. Hispanic or Latino refers to “A person of Cuban, Mexican, Puerto Rican, South or Central American, or other Spanish culture or origin, regardless of race” [30].

### 2.3. Statistical Analysis and Outcomes

The number and proportions of clinical trials reporting sex/gender, race, ethnicity, and city and province of Canadian clinical trial sites were calculated among all reported trials. The number and proportions of patients of each specific sex/gender, race, and ethnicity were calculated for each of the 25 clinical trials. Enrolment disparity for each sex/gender was calculated with two metrics: (1) the enrolment incidence disparity (EID), which measures enrolment disparity in absolute terms, and (2) the enrolment incidence ratio (EIR), which measures enrolment disparity for each sex/gender in relative terms [22]. The EID is calculated as the difference between the proportion of patients of a particular sex/gender among trial participants in the 25 clinical trials under study and the estimated proportion of patients of that particular sex/gender diagnosed with lung cancer among the Canadian population. EIR is calculated as the proportion of patients of a particular sex/gender among trial participants in the 25 clinical trials under study divided by the estimated proportion of patients of that particular sex/gender diagnosed with lung cancer among the Canadian population. The proportion of patients of a particular sex/gender diagnosed with lung cancer among the Canadian population is the person-based prevalence for lung and bronchus cancer by sex, with a prevalence duration of 5 years (2013–2018), as reported by the Canadian Cancer Statistics Dashboard [37]. The number and proportions of Canadian clinical trial sites were calculated per city and province. Lung cancer incidence rates per 100,000 person-years per province (2014) were reported per province, as well as daily/occasional smoking rate (%) (2015–2016) per province [11].

## 3. Results

### 3.1. Overview

A total of 25 lung cancer phase III clinical trials with Canadian sites and 16,783 evaluable participants were included in the review. There were 20 clinical trials for NSCLC and 5 trials for SCLC. Only one of the clinical trials was run exclusively in Canada (NCT02044380), whereas the other 24 clinical trials enrolled participants in other countries in addition to Canada. Out of the 25 clinical trials, 24 reported race and 18 also reported ethnicity as demographic characteristics (Figure 1). Eighteen clinical trials reported both race and ethnicity and six clinical trials reported race only. The exclusively Canada-based trial reported neither race nor ethnicity. All 25 clinical trials reported sex/gender data, and all reported the city as well as province or territory of the Canadian clinical trial sites. The basic characteristics of each clinical trial are included in Table 1.

Only six of the included clinical trials reported the number of participants in each country or region of enrolment. Three clinical trials reported the number of participants enrolled in the individual countries (e.g., Canada), and three others reported by broad regions (e.g., North America, East Asia vs. Non-East Asia). In the three multi-country clinical trials that disclosed participant enrolment in Canada, the proportion of Canadian participants ranged from 0.7% (2/275) to 3.3% (99/3014).

### 3.2. Sex/Gender Reporting and Representation

**Figure 2:**
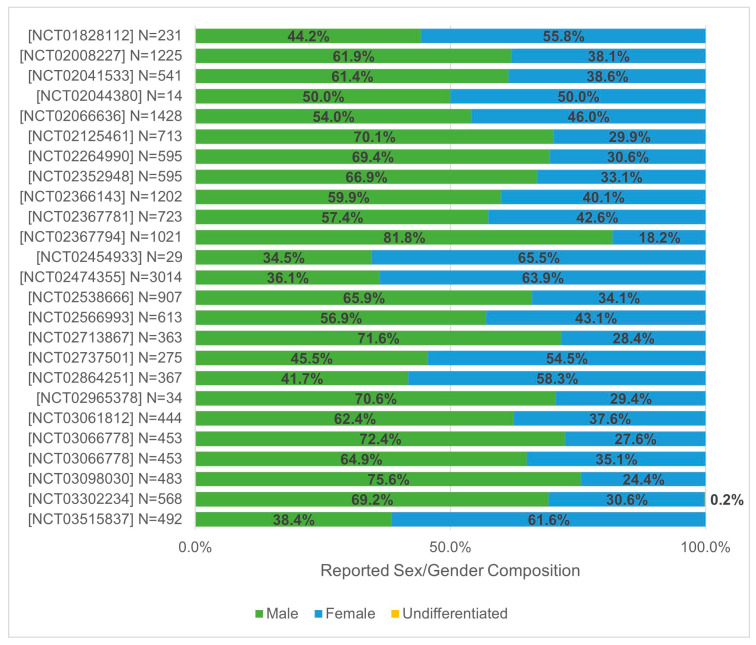
Sex/gender demographics of participants in lung cancer clinical trials with Canadian sites.

Figure 2 shows the sex/gender proportions for each of the included clinical trials. Out of the 25 clinical trials, 24 reported sex/gender as female and male, and 1 reported sex/gender as female, male, and undifferentiated. Most of the trials (18/25; 72.0%) had a higher male representation than female representation, six trials (24.0%) had a higher female representation, and the one trial (4.0%) that was exclusively based in Canada had equal male and female representation.

The total number of participants with sex/gender data was 16,783. Most of the participants were male (9703/16,783; 57.8%), followed by females (7079/16,783; 42.2%). One patient was undifferentiated (<1.0%).

**Figure 3:**
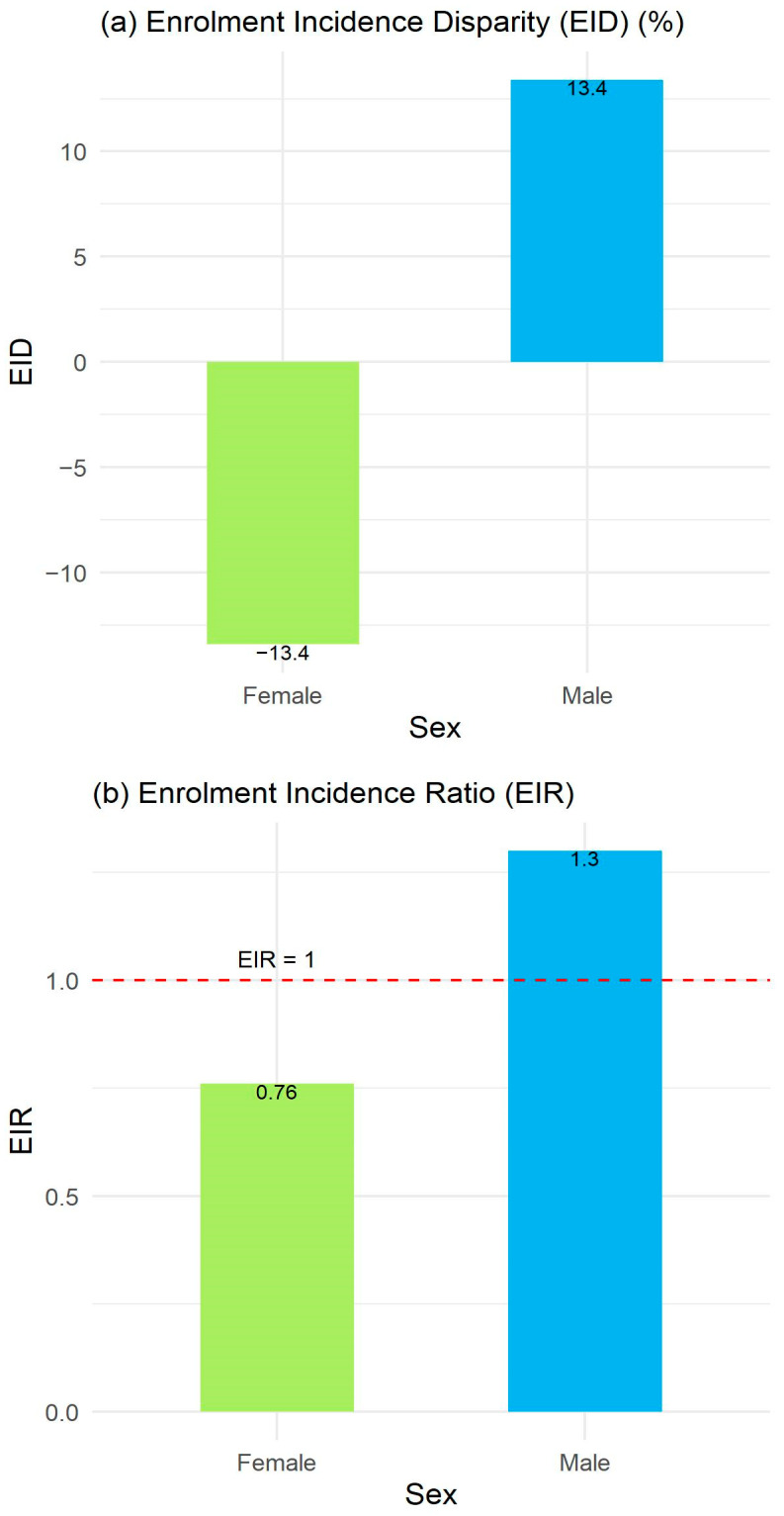
Enrolment to incidence disparity (EID) (**a**) and ratio (EIR) (**b**) by sex/gender.

Female participants were underrepresented (EID: −13.4%; EIR: 0.76, compared to EID = 13.4; EIR = 1.30 for males) compared with their expected proportions based on the incidence of lung cancer by sex in Canada (Figure 3). Since there were no missing sex/gender data, and the proportions of males and females were complementary and added up to 100%, the resulting EIDs were of equal magnitude but with opposite signs.

More than half of the trials (52.0%) included specific restrictions related to female participants of childbearing potential in their inclusion or exclusion criteria. Seven trials (28.0%) mentioned in the inclusion criteria that female participants of childbearing potential must be using contraception and/or have a negative pregnancy test. Nine trials (36.0%) excluded females who were pregnant or breastfeeding.

### 3.3. Reporting of Sex and Gender in Publications

The publications corresponding to the clinical trials (if any) were reviewed. There were publications associated with 21 out of the 25 included clinical trials (84%), and no publications were found for four of the clinical trials (16%). Among the publications for 21 of the clinical trials, all of them provided information about the sex or gender of participants. Two publications (9.5%) reported the number and/or percentage of males without specifying whether it referred to male sex or male gender [41,55]. One publication reported both male and female numbers and percentages under the category of gender [43], and another publication mentioned only the number and percentage of male gender [25]. None of the publications associated with the clinical trials mentioned how sex/gender data were collected.

### 3.4. Race and Ethnicity Reporting and Representation

The races reported for each clinical trial are shown in Table 2. Among the 24 clinical trials that reported race, the numbers of White as well as Black or African American participants were reported in 24 (100%) of the trials, Asian participation was reported in 23 (95.8%) trials, Unknown or not reported data were included in 21 (87.5%) trials, American Indian or Alaska Native and Native Hawaiian or Other Pacific Islander data were included in 19 (79.2%) trials. More than one race was represented in 16 (66.7%) trials, and Other participants were reported in eight trials (33.3%). There were 20 clinical trials (83.3%) with a White majority and four (16.7%) with an Asian majority.

The total number of participants across all clinical trials with race data was 16,769. For each of the clinical trials that reported race, the sum of participants reported under the cohort categories equaled the total number of eligible and evaluable participants, making all participant racial data accounted for. Most participants were White (11,083/16,769; 66.1%), followed by Asian (4740/16,769; 28.3%), Unknown or not reported (437/16,769; 2.6%), Black or African American (331/16,769; 2.0%), American Indian or Alaska Native (62/16,769; 0.4%), Other (57/16,769; 0.3%), More than one race (48/16,769; 0.3%), and Native Hawaiian or Other Pacific Islander (11/16,769; 0.1%).

The ethnicity demographics reported for each clinical trial are shown in Table 3. Out of the 18 clinical trials that reported ethnicity, all provided the number and proportion of participants under the Hispanic or Latino (or Spanish) category, 17 reported the number of Not Hispanic or Latino (or Spanish) participants, and 16 provided the number of participants with Unknown or not reported ethnicity. One trial reported solely under the Hispanic or Latino category and did not account for the remaining number of participants under the other cohort categories. There were 10,367 participants with a reported ethnicity. Most participants were Not Hispanic or Latino (8443/10,367; 81.4%), followed by Unknown or not reported (1321/10,367; 12.7%), and Hispanic or Latino (603/10,367; 5.8%).

### 3.5. Reporting of Race and Ethnicity in Publications

The publications for 17 of the included clinical trials with publications reported the race or ethnicity of the participants (17/21; 81.0%) [25,38,39,42,43,44,45,46,47,48,49,50,51,52,53,54,55,58,59,61]. One publication mentioned that some countries did not allow race data to be collected, and reported race under the categories of White, Black or African American, Asian, Other, and Not Available [52].

There were eight clinical trials with corresponding publications that reported ethnicity (8/21; 38.1%). For most of these clinical trials (5/8; 62.5%), the terms race and ethnicity were used interchangeably in the associated publications [25,38,45,46,48,52], and two reported ethnicity instead of race (2/8; 25.0%) [43,50]. In the publication for one clinical trial, the reported races were White and Asian, and ethnicity was categorized as Asian versus Non-Asian [49].

Only four publications stated how the race or ethnicity data were collected (4/21; 19.0%). Of these four publications, two stated that race or ethnicity was provided by the participants [42,45], and two stated that this was provided by the investigator [50,53]. Most of the papers were published in the New England Journal of Medicine (3/4; 75.0%) [42,45,53] and one was published in Future Oncology [50].

### 3.6. Geographical Location Reporting and Representation

A total of 51 unique clinical trial sites participated in Canada and some sites participated in more than one trial, resulting in a total of 117 sites across the 25 clinical trials. The clinical trials were only conducted in urban areas in Alberta, British Columbia, Manitoba, New Brunswick, Nova Scotia, Ontario, Quebec, and Saskatchewan. The province that hosted the most sites was Ontario (43.6%), followed by Quebec (34.2%), Alberta (8.5%), British Columbia (4.3%), New Brunswick (4.3%), Nova Scotia (2.6%), Manitoba (1.7%), and Saskatchewan (0.9%). The cities that hosted the most sites were Montreal (Quebec) and Toronto (Ontario) (14.5% each), followed by Oshawa (Ontario) (5.1%), Calgary (Alberta), Edmonton (Alberta), and Quebec City (Quebec) (4.3% each). The trial held exclusively in Canada was only conducted at four sites in Ontario and one site in Quebec (Montreal). Furthermore, out of the 25 trials included in the literature search, only the trial that was conducted exclusively in Canada did not include the postal codes for the sites. The top three postal codes that were referenced the most frequently were associated with centers in Toronto (10/25 trials; 40.0%), Calgary (5/25 trials; 20.0%), and Edmonton (5/25 trials; 20.0%).

**Figure 4:**
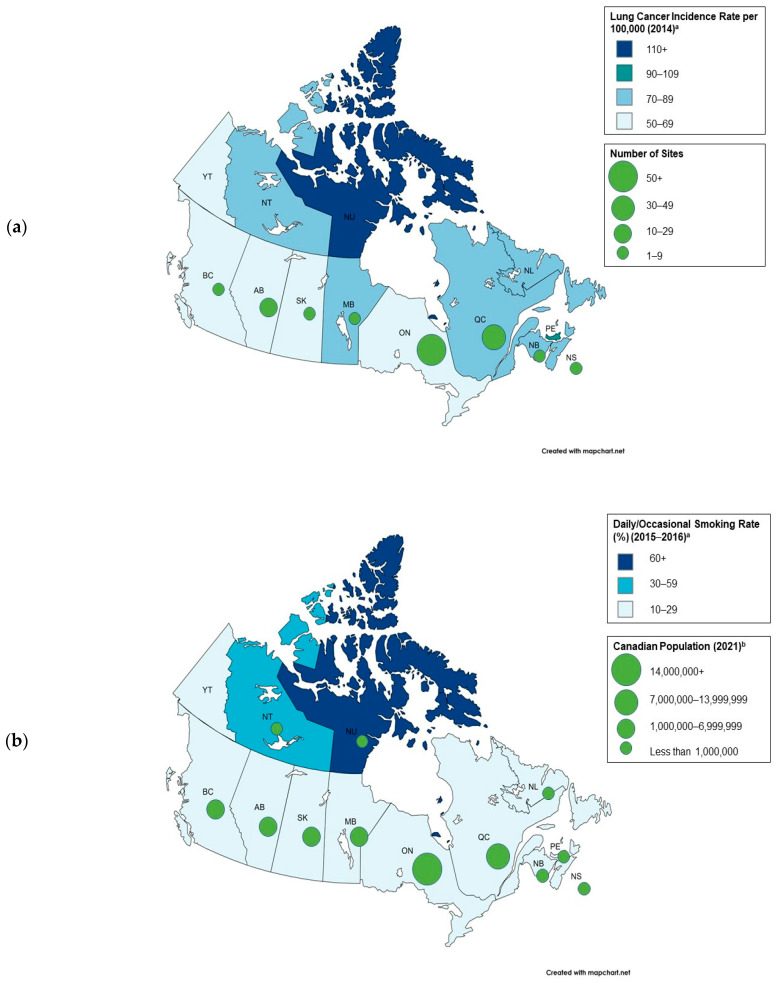
Geographical variation in (**a**) lung cancer incidence rate and number of clinical trial sites, and (**b**) daily/occasional smoking rate and Canadian population. AB: Alberta; BC: British Columbia; MB: Manitoba; NB: New Brunswick; NL: Newfoundland and Labrador; NT: Northwest Territories; NS: Nova Scotia; NU: Nunavut; ON: Ontario; PE: Prince Edward Island; QC: Quebec; SK: Saskatchewan; YT: Yukon. ^a^ The lung cancer incidence rates for 2014 and daily/occasional smoking rates for 2015–2016 were obtained from the Canadian Partnership Against Cancer’s 2018 Cancer System Performance Report [11]. ^b^ The 2021 Canadian population amounts were obtained from Statistics Canada’s 2021 Census of Population Profile [62].

Figure 4 shows the geographic variation in lung cancer incidence rates, smoking rate, Canadian population, and number of sites by province and territory. Nunavut and the Northwest Territories have some of the highest lung cancer incidence rates and smoking rates but did not host any sites.

## 4. Discussion

### 4.1. Sex/Gender Reporting and Representation

This review assessed how geographic and sociodemographic characteristics are reported in lung cancer clinical trials with Canadian participation and examined how different groups are represented relative to the Canadian population. Reporting of participant sex/gender data within the clinical trials was mostly completed under the categories of female and male, but there is a lack of transparency regarding the data collection method used to determine sex/gender. It was unknown if the data were based on self-reporting, genomics, or alternative methods. Furthermore, only one clinical trial reported participant sex/gender with a category other than male or female, with a single individual identifying as undifferentiated. The rest of the trials all reported the sex/gender of participants as male or female. This highlights a lack of gender reporting and inclusion in clinical trials. Considering that individuals who identify as gender minorities may be disproportionally impacted by lung cancer due to higher smoking prevalence [13], the reporting of gender-related data could facilitate the collection of information essential for promoting equity, diversity, and inclusion in clinical trials.

Lung cancer incidence rates are slightly higher in males compared to females in Canada [4,5] but for most of the included clinical trials, male representation was generally greater than female representation. Overall, representation was higher for males relative to their estimated proportions among the Canadian lung cancer population. A study comparing gender representation in clinical trials and the United States population found an EID of 3.2 for lung and bronchus cancer [63]. Although the gender representation in lung and bronchus cancer clinical trials reported by the US study (59.1% males, 40.9% females) was similar to what was observed in the current review (57.8% males, 42.2% females), the population-based estimates by sex/gender differed between the United States and Canada [63]. Thus, it is important that Canadian healthcare professionals and researchers keep the Canadian lung cancer population in mind when reviewing results from global clinical studies.

More than half of the included clinical trials had inclusion and exclusion criteria limiting conditions traditionally associated with females, such as pregnancy and breastfeeding. However, this may not have been a primary reason for the low female representation, as lung cancer is most commonly diagnosed in people aged 50 or older (98% of all cases) [4]. The lower female representation in clinical trials may have been due to other factors such as restraints due to familial responsibilities (e.g., lack of time) or hesitancy by researchers to approach potential female participants due to gender-based misconceptions or concerns regarding toxicity and tolerability [64,65]. As such, greater effort is needed from researchers and sites to identify and approach eligible female participants. Researchers should investigate the reasons behind the underrepresentation of women in clinical trials and devise strategies to address the issues. For instance, quotas could be implemented to encourage the recruitment of more balanced proportions of men and women participants. It is recommended that researchers consider the impact of sex/gender throughout the clinical trial process and implement the SAGER guidelines [27].

### 4.2. Race and Ethnicity Reporting and Representation

Most of the clinical trials were consistent in their reporting of race and/or ethnicity on ClinicalTrials.gov and followed the NIH/OMB classification categories [30]. However, it is difficult to interpret the findings for the Canadian population as these categories are not consistent with those used by Statistics Canada to report on population groups, which include Arab, Black, Chinese, Filipino, Indigenous Identity (First Nations, Inuit, and Métis), Japanese, Korean, Latin American, South Asian (such as East Indian, Pakistani, and Sri Lankan), Southeast Asian (such as Vietnamese, Cambodian, Laotian, and Thai), West Indian (such as Iranian and Afghan), and White [66]. For instance, the NIH/OMB category of White includes people with origins in Europe, the Middle East, or North Africa [30], but Arab is considered a separate population group in Canada and would not fall under the category of White [66]. Moreover, the NIH/OMB classification categories inadequately represent Canadian Indigenous populations as they may report under American Indian or Alaska Native, or simply as Other. This would fail to illustrate data specific to the Indigenous populations within Canada. Overall, with the lack of more specific categories, conclusions drawn from clinical trial data may be overly simplistic and too generalized. In March 2024, the OMB updated the race and ethnicity reporting standards for federal data and introduced a new Middle Eastern or North African category [67]. However, it remains uncertain whether or when this new category will be adopted by ClinicalTrials.gov and if trial designs and data collection will be updated.

Furthermore, genomic alterations that serve as targets for novel therapeutics have been observed at varying rates across different racial/ethnic groups [68]. This highlights the importance of reporting subpopulations, such as East Asian rather than Asian. In addition, being of a mixed racial and ethnic background can influence genomics, but the precise racial or ethnic makeup of cohorts identified as “More than one race”, as well as “Other” is usually not reported.

When comparing the extracted clinical trial data with lung cancer incidence rates within Canada, Indigenous Peoples of Canada, who may have been captured as American Indian or Alaska Native, or Other, and Black or African American cohorts were noted as underrepresented groups among the clinical trials. The underrepresentation of these cohorts may be due to the low population size in the participating countries, as well as the lack of access to healthcare, including physician care and genomic testing [69,70,71]. This further contributes to a lack of recorded lung cancer rates, genomic data, and racial and ethnic identifiers in databases, ultimately resulting in insufficient specialist care and clinical trial participation. In addition, mistrust due to past research injustices and the fear of discrimination and racism may contribute to underrepresentation and lack of participant retention within clinical trials [69,70,71]. White and Asian were the highest-represented cohorts, possibly due to the geographic locations of the clinical trials (e.g., Asian countries like South Korea and Taiwan) or their greater access to care and availability of genomic data compared to other population groups [72]. Native Hawaiian or other Pacific Islander was the lowest reported cohort, possibly due to their low populations in the participating countries compared to the other cohorts. Efforts should be made to ensure greater representation for this group in areas known to have significant proportions of Hawaiian or Pacific Islander populations.

Although the consistent reporting of cohort categories among the clinical trials helped to draw some conclusions from the data, there were several limitations of the available data. For instance, only a few of the trials provided details on how race and ethnicity demographic information was collected. Most trials had participants in the Unknown or not reported cohort, and some had a sizeable proportion of participants within this category. Some reasons for a large number of “Unknown or not reported” include lack of race or ethnicity information in patient records, legal restrictions as some countries do not permit the collection of race data (e.g., France, Italy) [73], reluctance among participants to disclose this information due to fear of discrimination or privacy concerns [74], or lack of representation among the provided race and ethnicity categories [74]. Moreover, it remains uncertain whether additional details regarding race and ethnicity demographics, including subpopulation data, were obtained but not published externally. The lack of publicly available data limits the public’s ability to assess racial and ethnic representation. Insufficient publicly available data on the lung cancer population in Canada broken down by racial categories prevented the calculation of EID and EIR by race. For this review, EID and EIR could only be calculated for sex/gender. The only Canada-based clinical trial included in the review failed to report any race and ethnicity demographic information (NCT02044380), underscoring the need to improve reporting standards for demographic data in Canada and increase the transparency of data to monitor representativeness. Including such data in public databases like ClinicalTrials.gov and Health Canada’s Clinical Trial is important in reducing the difficulties associated with data access. However, data obtained from surveys may be subject to restrictions to protect the confidentiality of survey respondents [6].

### 4.3. Geographic Location Reporting and Representation

As only six of the included clinical trials reported the number of participants by region of enrolment, assessing how many participants were included from Canada in each clinical trial is challenging. In addition, participant race and ethnicity by clinical trial site were not reported, which limited the ability to assess demographic reporting and representation by research sites based in Canada. When assessing the extracted clinical trial data with respect to lung cancer incidence rates across provinces/territories, the density of site locations per province/territory often did not reflect lung cancer incidence rates by province/territory. Overrepresented provinces/territories included Ontario and Quebec, despite not having the highest lung cancer incidence rates compared to other provinces/territories [8]. Similarly, the exclusively Canada-based clinical trial included in the review (NCT02044380) was conducted primarily in Ontario, with one location in Quebec. Nunavut, the Northwest Territories, Yukon, Newfoundland and Labrador, and Prince Edward Island did not host any sites in any of the trials. It is especially important to address the lack of representation in Nunavut, the Northwest Territories, Yukon, and Newfoundland and Labrador residents, as they are particularly vulnerable due to their high smoking rates and vast geographic distances to other research sites [8]. Moreover, given that Indigenous Peoples constitute the majority of Canada’s population in Nunavut, the Northwest Territories, and Yukon, it is essential to incorporate methods to enhance their representation in the design of clinical trials conducted in Canada [9].

The Canadian sites used for the clinical trials included in this study were all located in urban areas, possibly due to increased resource availability and access to personnel to successfully run a clinical trial. This presents major accessibility barriers to clinical trial participation for those living in rural and remote areas, although major cities may offer a range of transportation options from a variety of locations. One limitation is the absence of data on the geographic locations of the participants of the included trials, making it unclear whether participants from rural and remote areas may have traveled to the urban centers for care. Remote and rural areas tend to experience higher lung cancer incidence rates [15], and as Indigenous Peoples are more likely to live in these areas, they are particularly vulnerable [14].

### 4.4. Future Directions

The findings of this review highlight the need to improve diversity and demographic representation in clinical trials. There is a need for racially disaggregated health data in Canada, similar to the United States [6], and Health Canada has introduced a new questionnaire for regulatory submissions to address this gap [31,32]. Additionally, it should be required for clinical trials to be included in a registry or report demographic and geographic information (e.g., number of participants recruited per location). Currently, Health Canada recommends that sponsors register on ClinicalTrials.gov or the International Standard Registered Clinical/Social Study Number Registry (ISRCTN), and although there are some Canadian databases and registries, such as the Clinical Trials Database and Canadian Cancer Trials, not all Canadian clinical trials are registered on these [75]. The Canadian Cancer Registry (CCR) collects incidence data by age, province, and sex, but not by race and ethnicity [6]. Thus, insights on demographic representation in clinical trials rely on data-linkage studies involving the Canadian Cancer Registry and other databases [6].

Starting in 2017, ClinicalTrials.gov requires that race and/or ethnicity information be reported [27,29]. However, the current race and ethnicity reporting standards are suitable for the United States population and should be expanded further to ensure applicability across different countries. The guidance provided by CIHI can be adapted [33,76]. For example, the race categories of Asian and White could be further subdivided into distinct subpopulations, such as East Asian, South Asian, and Middle Eastern. If participants identify as mixed race, they can be asked to select all categories that apply. If participants identify as Other, they can be asked to specify. Recently, the OMB introduced a new Middle Eastern or North African category and combined race and ethnicity into one question in an effort to reduce the number of respondents selecting Some Other Race [67]. Although these changes in race and ethnicity reporting standards have been issued for federal data [67], they should also be adopted by ClinicalTrials.gov and the data collection forms used for clinical trials. Specific ethnicity-related data could also be collected, such as language, religion, and country of birth [76]. It is important to consider whether the options provided are appropriate for the region where the clinical trial is taking place and encourage self-reporting by participants [77]. Furthermore, researchers should avoid using the terms “race” and “ethnicity”, as well as “sex” and “gender”, interchangeably as they are not synonymous [35,36,38,78]. If one term is substituted for the other, clear definitions of the terms should be provided [78].

Moreover, there needs to be more careful consideration of clinical trial site locations and greater efforts to ensure that research participants are representative of the population that will be treated. There should be transparency on the number of clinical trial participants that are enrolled in Canada, and in the context of lung cancer, further attempts should be made to recruit participants from regions with high incidence of disease, such as Nunavut and Atlantic Canada [8]. The lack of infrastructure in rural and remote locations can be addressed by collaborating with reputable institutions and leveraging existing sites to set up decentralized clinical trials [79,80]. Additionally, various factors such as an individual’s cultural beliefs, health literacy, socioeconomic status, view of Western medicine, and trust or mistrust towards the healthcare system may impact their receptiveness towards new treatments and clinical trials [70]. Researchers and sponsors should educate themselves and involve the populations of interest in clinical trial set-up and decision-making processes. They should take into consideration the priorities and perspectives of the populations of interest when designing the trial. For example, traditional Indigenous ways of healing, knowledge, and approach to health need to be respected and considered when working with Indigenous Peoples [81]. As Indigenous health is community-centered, it is important that researchers discuss the community impact of the clinical trial and the collective ownership of research data with potential participants [81]. Diversity among the investigators conducting the clinical research should also be encouraged [80].

Clinical trial education and community outreach may help to spread awareness of clinical trials and build trust with underrepresented populations, especially those who have faced mistreatment in health research [80]. Participants should be made aware of the purpose behind collecting demographic information (e.g., to assess diversity and demographic representation in the clinical trial) and how their data will be safeguarded [76]. It is important to emphasize that the disclosure of this information will not impact their care [76]. Healthcare professionals and staff delegated to collecting demographic information need to be trained on the importance of adequate representation of minority groups and females in clinical trials and accurate reporting of demographic information [77].

## 5. Conclusions

Ensuring diverse demographic representation and accurate reporting in clinical trials is crucial for improving the health outcomes of Canada’s lung cancer population. Groups that must be prioritized in Canada include, but are not limited to, the Indigenous Peoples of Canada, and individuals living in rural or remote areas. All those involved in clinical trials, including clinical trial regulators, must ensure their involvement is being viewed through an equity, diversity, and inclusion lens for improvements to be made. This includes looking at the social determinants of health that pose barriers to accessing clinical trials for underrepresented and disadvantaged groups. Additional focus should be brought to establishing consistent demographic reporting standards and practices that reflect the diversity and uniqueness of people, as well as transparency of such data. This may include amending standards nationally and advocating for change internationally. There is both a scientific and ethical stake in ensuring adequate demographic reporting and representation in clinical trials.

## Figures and Tables

**Table 1:** Clinical trial characteristics including the trial phase, medical condition, trial start and completion dates, trial focus, and participating countries for each trial included in the review.

Trial Ref. ^a^	NCT ID ^b^	Phase	Medical Condition	Cancer Stage	Trial Start Date	Trial Completion Date	Trial Focus	Intervention(s)	Total Number of Evaluable Participants	Participating Countries (n) ^c^
[38]	NCT01828112	3	NSCLC	IIIB or IV	2013-06	2023-11	Drug trial	Ceritinib, Docetaxel, Pemetrexed	231	Belgium, **Canada**, France, Germany, Hong Kong, Ireland, Israel, Italy, Japan, Korea (Republic of), Lebanon, Netherlands, Portugal, Russian Federation, Singapore, Spain, Switzerland, Turkey, United Kingdom, United States (20)
[39]	NCT02008227	3	NSCLC	IIIB, IV, or recurrent	2014-03	2019-01	Drug trial	Atezolizumab, Docetaxel	1225	Argentina, Austria, Brazil, **Canada**, Chile, Finland, France, Germany, Greece, Guatemala, Hungary, Italy, Japan, Korea (Republic of), Netherlands, New Zealand, Norway, Panama, Poland, Portugal, Russian Federation, Serbia, Spain, Sweden, Switzerland, Taiwan, Thailand, Turkey, Ukraine, United Kingdom, United States (31)
[40]	NCT02041533	3	NSCLC	IV or recurrent	2014-03	2022-05	Drug trial	Carboplatin, Cisplatin, Gemcitabine, Nivolumab, Paclitaxel, Pemetrexed	541	Argentina, Australia, Austria, Belgium, Brazil, **Canada**, Czechia, Finland, France, Germany, Greece, Hungary, Italy, Japan, Korea (Republic of), Mexico, Netherlands, Poland, Romania, Spain, Sweden, Switzerland, Taiwan, Turkey, United Kingdom, United States (26)
NA	NCT02044380	3	NSCLC	III or IV	2014-03	2016-02	Drug trial	Afatinib	14	**Canada (1)**
[41]	NCT02066636	3	NSCLC	IIIB, IV, or recurrent	2014-04	2021-10	Drug trial	Nivolumab	1428	**Canada**, United States (2)
[42]	NCT02125461	3	NSCLC	III	2014-05	2023-08	Drug trial	Durvalumab	713	Australia, Belgium, **Canada**, Chile, France, Germany, Greece, Hungary, Israel, Italy, Japan, Korea (Republic of), Mexico, Netherlands, Peru, Poland, Singapore, Slovakia, South Africa, Spain, Taiwan, Thailand, Turkey, United Kingdom, Vietnam, United States (26)
[43]	NCT02264990	3	NSCLC	III or IV	2014-09	2020-02	Drug trial	Carboplatin, Cisplatin, Paclitaxel, Pemetrexed, Veliparib	595	Argentina, Australia, **Canada**, Czechia, Denmark, Finland, Germany, Hungary, Israel, Japan, Korea (Republic of), Netherlands, New Zealand, Russian Federation, South Africa, Spain, Taiwan, Turkey, United Kingdom, United States (20)
[44]	NCT02352948	3	NSCLC	IIIB or IV	2015-01	2023-08	Drug trial	Durvalumab, Durvalumab in combination with Tremelimumab, Erlotinib, Gemcitabine, Tremelimumab, Vinorelbine	595	Australia, Belgium, Bulgaria, **Canada**, Chile, Czechia, France, Germany, Greece, Hong Kong, Hungary, Israel, Italy, Japan, Korea (Republic of), Netherlands, Poland, Romania, Russian Federation, Serbia, Singapore, Spain, Taiwan, Thailand, United Kingdom, United States (26)
[45,46]	NCT02366143	3	NSCLC	IV	2015-03	2020-12	Drug trial	Atezolizumab, Bevacizumab, Carboplatin, Paclitaxel	1202	Argentina, Australia, Austria, Belgium, Brazil, Bulgaria, **Canada**, Chile, France, Germany, Italy, Japan, Latvia, Lithuania, Mexico, Netherlands, Peru, Portugal, Russian Federation, Singapore, Slovakia, Spain, Switzerland, Taiwan, Ukraine, United States (26)
[47]	NCT02367781	3	NSCLC	IV	2015-04	2021-01	Drug trial	Atezolizumab, Carboplatin, Nab-Paclitaxel, Pemetrexed	723	Belgium, **Canada**, France, Germany, Israel, Italy, Spain, United States (8)
[48]	NCT02367794	3	NSCLC	IV	2015-06	2021-02	Drug trial	Atezolizumab, Carboplatin, Nab-Paclitaxel, Paclitaxel	1021	Argentina, Australia, Austria, Belgium, Brazil, Bulgaria, **Canada**, Chile, France, Germany, Israel, Italy, Japan, Latvia, Lithuania, Mexico, Netherlands, Peru, Portugal, Russian Federation, Singapore, Slovakia, Spain, Taiwan, Ukraine, United States (26)
[49]	NCT02454933	3	NSCLC	III or IV	2015-07	2023-06	Drug trial	Durvalumab, Osimertinib	29	**Canada**, Korea (Republic of), Taiwan (3)
[50]	NCT02474355	3	NSCLC	IIIB or IV	2015-09	2019-04	Drug trial	Osimertinib	3014	Argentina, Australia, Austria, Belgium, Brazil, **Canada**, China, Denmark, Ireland, Italy, Korea (Republic of), Saudi Arabia, Spain, Sweden, Taiwan, United Kingdom (16)
[51]	NCT02538666	3	SCLC	Extensive stage	2015-10	2021-11	Drug trial	Ipilimumab, Nivolumab	907	Argentina, Australia, Austria, Belgium, Brazil, **Canada**, China, Colombia, Finland, France, Germany, Greece, Hong Kong, Ireland, Israel, Italy, Japan, Korea (Republic of), Mexico, Netherlands, Peru, Poland, Romania, Russian Federation, Singapore, South Africa, Spain, Sweden, Switzerland, Taiwan, United Kingdom, United States (32)
[52]	NCT02566993	3	SCLC	Limited or extensive stage	2016-08	2020-02	Drug trial	Cyclophosphamide, Doxorubicin, Lurbinectedin, Topotecan, Vincristine	613	Argentina, Austria, Belgium, Brazil, Bulgaria, **Canada**, Czechia, France, Germany, Greece, Hungary, Italy, Lebanon, Netherlands, Poland, Portugal, Romania, Spain, United Kingdom, United States (20)
NA	NCT02713867	3	NSCLC	IIIB or IV	2016-05	2022-01	Drug trial	Nivolumab	363	Australia, Austria, **Canada**, France, Germany, Italy, Spain, United States (8)
[53,54]	NCT02737501	3	NSCLC	IIIB or IV	2016-05	2021-01	Drug trial	Brigatinib, Crizotinib	275	Australia, Austria, **Canada**, Denmark, France, Germany, Hong Kong, Italy, Korea (Republic of), Luxembourg, Netherlands, Norway, Singapore, Spain, Sweden, Switzerland, Taiwan, United Kingdom, United States (19)
[55]	NCT02864251	3	NSCLC	IV	2017-03	2022-10	Drug trial	Carboplatin, Cisplatin, Ipilimumab, Nivolumab, Pemetrexed	367	**Canada**, China, France, Hong Kong, Japan, Korea (Republic of), Singapore, Spain, Taiwan, United States (10)
[25]	NCT02965378	2/3	NSCLC	IV	2014-10	2019-10	Drug trial	Docetaxel, Fexagratinib	34	**Canada**, United States (2)
NA	NCT03061812	3	SCLC	Extensive stage	2017-04	2020-02	Drug trial	Dexamethasone, Rovalpituzumab tesirine, Topotecan	444	Australia, Belarus, Belgium, Brazil, Bulgaria, **Canada**, China, Croatia, Czechia, Denmark, France, Germany, Greece, Hungary, Italy, Japan, Korea (Republic Of), Latvia, Mexico, Netherlands, Poland, Portugal, Romania, Russian Federation, Serbia, Singapore, Spain, Sweden, Taiwan, Turkey, Ukraine, United Kingdom, United States (33)
[56,57]	NCT03066778	3	SCLC	Extensive stage	2017-05	2021-09	Drug trial	Carboplatin, Cisplatin, Etoposide, Pembrolizumab	453	Australia, **Canada**, Chile, France, Germany, Hungary, Ireland, Israel, Japan, Korea (Republic of), New Zealand, Poland, Russian Federation, Spain, Switzerland, Taiwan, Turkey, United Kingdom, United States (19)
[58]	NCT03098030	2/3	SCLC	NR	2017-06	2020-03	Drug trial	Dinutuximab, Irinotecan, Topotecan	483	Australia, Bulgaria, **Canada**, France, Georgia, Hong Kong, Hungary, India, Italy, Korea (Republic of), Lithuania, Malaysia, Philippines, Poland, Romania, Russian Federation, Slovakia, Spain, Taiwan, Thailand, Ukraine, United Kingdom, United States (23)
[59]	NCT03191786	3	NSCLC	IIIB or IV	2017-09	2023-10	Drug trial	Atezolizumab, Gemcitabine, Vinorelbine	453	Argentina, Belgium, Brazil, Bulgaria, **Canada**, China, Colombia, Czechia, Denmark, Germany, India, Ireland, Italy, Kazakhstan, Luxembourg, Mexico, Poland, Portugal, Romania, Slovakia, Spain, Switzerland, United Kingdom, Vietnam (24)
[60]	NCT03302234	3	NSCLC	IV	2017-12	2022-09	Drug trial	Ipilimumab, Pembrolizumab	568	Argentina, Australia, Brazil, **Canada**, Chile, Colombia, France, Germany, Hungary, Ireland, Italy, Korea (Republic of), Latvia, Mexico, Peru, Poland, South Africa, Spain, Taiwan, Thailand, Turkey, Ukraine, United Kingdom, United States (24)
NA	NCT03515837	3	NSCLC	IV	2018-06	2023-10	Drug trial	Carboplatin, Cisplatin, Pembrolizumab, Pemetrexed	492	Australia, Brazil, **Canada**, China, France, Germany, Hong Kong, Israel, Italy, Japan, Korea (Republic of), Mexico, Spain, Sweden, Taiwan, United Kingdom, United States (17)

NA: not available; NSCLC: non-small-cell lung cancer; NR: not reported; SCLC: small-cell lung cancer. ^a^ Trial Ref. refers to the publication(s) associated with the clinical trial. NA was used if there was no associated publication. ^b^ The ClinicalTrials.gov pages for the clinical trials were last accessed on 1 March 2024. ^c^ The total number of participating countries for each included clinical trial is included in parentheses, and Canada is shown in bold font.

**Table 2:** Race demographic characteristics of participants of lung cancer clinical trials with Canadian sites (**a**) and description of colors used in the heatmap table (**b**).

(a)											
NCT ID ^a^	Number of Participating Countries	Total Number of Evaluable Participants	Demographic Count, n (%) ^b^	White, n (%)	Black or African American, n (%)	Asian, n (%)	American Indian or Alaska Native, n (%)	Native Hawaiian or Other Pacific Islander, n (%)	More than One Race, n (%)	Other, n (%)	Unknown or Not Reported, n (%)
NCT01828112	20	231	231 (100)	149 (64.5) ^c^	1 (0.4)	68 (29.4)	NR	NR	NR	6 (2.6)	7 (3.0)
NCT02008227	31	1225	1225 (100)	870 (71.0)	27 (1.4)	249 (20.3)	3 (0.2)	5 (0.4)	3 (0.2)	23 (1.9)	45 (3.7)
NCT02041533	26	541	541 (100)	470 (86.9)	16 (3.0)	47 (8.7)	1 (0.2)	0 (0.0)	NR	7 (1.3)	NR
NCT02044380	1	14	-	NR	NR	NR	NR	NR	NR	NR	NR
NCT02066636	2	1428	1428 (100)	1266 (88.7)	105 (7.4)	42 (2.9)	5 (0.4)	2 (0.1)	0 (0.0)	NR	8 (0.6)
NCT02125461	26	713	713 (100)	494 (69.3)	14 (2.0)	192 (26.9)	9 (1.3)	2 (0.3)	NR	1 (0.1)	1 (0.1)
NCT02264990	20	595	595 (100)	462 (77.6)	22 (7.4)	110 (18.5)	NR	NR	NR	1 (0.2)	NR
NCT02352948	26	595	595 (99.7)	405 (68.1)	9 (1.5)	177 (29.7)	0 (0.0)	0 (0.0)	NR	3 (0.5)	1 (0.2)
NCT02366143	26	1202	1202 (100)	988 (82.2)	24 (2.0)	150 (12.5)	4 (0.3)	0 (0.0)	7 (0.6)	NR	29 (2.4)
NCT02367781	8	723	723 (100)	650 (89.9)	26 (3.6)	17 (2.4)	0 (0.0)	0 (0.0)	2 (0.3)	NR	28 (3.9)
NCT02367794	26	1021	1021 (100)	869 (85.1)	14 (1.4)	112 (11.0)	5 (0.5)	1 (0.1)	8 (0.8)	NR	12 (1.2)
NCT02454933	3	29	29 (100)	3 (10.3)	0 (0.0)	26 (89.7)	0 (0.0)	0 (0.0)	0 (0.0)	NR	0 (0.0)
NCT02474355	16	3014	3014 (100)	897 (29.8)	21 (0.7)	2085 (69.2)	0 (0.0)	0 (0.0)	0 (0.0)	NR	11 (0.4)
NCT02538666	32	907	907 (100)	627 (69.1)	9 (1.0)	258 (28.4)	NR	NR	NR	12 (1.3)	1 (0.1)
NCT02566993	20	613	613 (100)	531 (86.6)	2 (0.3)	1 (0.2)	NR	NR	NR	4 (0.7)	75 (12.2)
NCT02713867	8	363	363 (100)	337 (92.8)	15 (4.1)	4 (1.1)	0 (0.0)	0 (0.0)	0 (0.0)	NR	7 (1.9)
NCT02737501	19	275	275 (100)	162 (58.9)	2 (0.7)	108 (39.3)	0 (0.0)	0 (0.0)	0 (0.0)	NR	3 (1.1)
NCT02864251	10	367	367 (100)	21 (5.7)	0 (0.0)	343 (93.5)	0 (0.0)	0 (0.0)	0 (0.0)	NR	3 (0.8)
NCT02965378	2	34	34 (79.1)	30 (88.2)	4 (11.8)	NR	NR	NR	NR	NR	NR
NCT03061812	33	444	444 (100)	358 (80.6)	2 (0.5)	80 (18.0)	1 (0.2)	1 (0.2)	2 (0.5)	NR	0 (0.0)
NCT03066778	19	453	453 (100)	339 (74.8)	1 (0.2)	86 (19.0)	0 (0.0)	0 (0.0)	1 (0.2)	NR	26 (5.7)
NCT03098030	23	483	483 (100)	277 (57.3)	6 (1.2)	80 (16.6)	0 (0.0)	0 (0.0)	1 (0.2)	NR	119 (24.6)
NCT03191786	24	453	453 (100)	298 (65.8)	3 (0.7)	113 (24.9)	21 (4.6)	0 (0.0)	12 (2.6)	NR	6 (1.3)
NCT03302234	24	568	568 (100)	441 (77.6)	1 (0.2)	64 (11.3)	7 (1.2)	0 (0.0)	11 (1.9)	NR	44 (7.7)
NCT03515837	17	492	492 (100)	139 (28.3)	7 (1.4)	328 (66.7)	6 (1.2)	0 (0.0)	1 (0.2)	NR	11 (2.2)
**(b)**											
**Color**	**Description**										
	Percentage values in the third quartile up to the maximum value for the trial										
	Percentage values between the median and third quartile for the trial										
	Percentage values between the first quartile and median for the trial										
	Percentage values from the first quartile down to the lowest value for the trial										
	Not reported (NR) is left unshaded										

NR: not reported. Four shades of blue represent the distribution of participants by race within each trial, by quartiles. NR is left unshaded. Table 2b describes the colors used in the heatmap table. ^a^ The ClinicalTrials.gov pages for the clinical trials were last accessed on 1 March 2024. ^b^ Demographic count refers to the number of participants with race data. The percentage for each cohort category for each clinical trial was calculated with the demographic count as the denominator. ^c^ The trial used the category Caucasian, and the number for this category was documented under White in this table.

**Table 3:** Ethnicity demographic characteristics of participants of lung cancer clinical trials with Canadian sites (**a**) and description of colors used in the heatmap table (**b**).

(a)						
NCT ID ^a^	Number of Participating Countries	Total Number of Evaluable Participants	Demographic Count, n (%) ^b^	Hispanic or Latino, n (%)	Not Hispanic or Latino, n (%)	Unknown or Not Reported, n (%)
NCT01828112	20	231	-	NR	NR	NR
NCT02008227	31	1225	1225 (100)	90 (7.3)	1081 (88.2)	54 (4.4)
NCT02041533	26	541	541 (100)	7 (1.3)	280 (51.8)	254 (47.0)
NCT02044380	1	14	-	NR	NR	NR
NCT02066636	2	1428	1428 (100)	41 (2.9)	1381 (96.7)	6 (0.4)
NCT02125461	26	713	-	NR	NR	NR
NCT02264990	20	595	595 (100)	48 (8.1)	547 (91.9)	0 (0.0)
NCT02352948	26	595	-	NR	NR	NR
NCT02366143	26	1202	-	NR	NR	NR
NCT02367781	8	723	723 (100)	37 (5.1)	639 (88.4)	47 (6.5)
NCT02367794	26	1021	1021 (100)	79 (7.7)	902 (88.3)	40 (3.9)
NCT02454933	3	29	29 (100)	1 (3.4)	28 (96.6)	0 (0.0)
NCT02474355	16	3014	-	NR	NR	NR
NCT02538666	32	907	907 (100)	40 (4.4)	495 (54.6)	372 (41.0)
NCT02566993	20	613	-	NR	NR	NR
NCT02713867	8	363	363 (100)	7 (1.9)	233 (64.2)	123 (33.9)
NCT02737501	19	275	275 (100)	16 (5.8) ^c^	259 (94.2) ^d^	NR
NCT02864251	10	367	367 (100)	0 (0.0)	171 (46.6)	196 (53.4)
NCT02965378	2	34	2 (5.9)	2 (5.9)	NR	NR
NCT03061812	33	444	444 (100)	11 (2.5)	433 (97.5)	0 (0.0)
NCT03066778	19	453	453 (100)	19 (4.2)	396 (87.4)	38 (8.4)
NCT03098030	23	483	483 (100)	11 (2.3)	355 (73.5)	117 (24.2)
NCT03191786	24	453	453 (100)	70 (15.5)	367 (81.0)	16 (3.5)
NCT03302234	24	568	568 (100)	91 (16.0)	433 (76.2)	44 (7.7)
NCT03515837	17	492	492 (100)	35 (7.1)	443 (90.0)	14 (2.8)
**(b)**						
**Colour**	**Description**					
	Percentage values in the third quartile up to the maximum value for the trial					
	Percentage values between the median and third quartile for the trial					
	Percentage values between the first quartile and median for the trial					
	Percentage values from the first quartile down to the lowest value for the trial					
	Not reported (NR) is left unshaded					

NR: not reported. Four shades of blue represent the distribution of participants by ethnicity within each trial, by quartiles. NR is left unshaded. Table 3b describes the colors used in the heatmap table. ^a^ The ClinicalTrials.gov pages for the clinical trials were last accessed on 1 March 2024. ^b^ Demographic count refers to the number of participants with race data. The percentage for each cohort category for each clinical trial was calculated with the demographic count as the denominator, except for NCT02965378, which only reported the number of Hispanic or Latino participants. ^c^ The trial reported Hispanic, Latino, or Spanish, and the numbers for this category are documented under Hispanic or Latino in this table. ^d^ The trial reported Not Hispanic, Latino, or Spanish, and the numbers for this category are documented under Not Hispanic or Latino in this table.

## Data Availability

The data analyzed in this study were obtained from ClinicalTrials.gov at https://clinicaltrials.gov/ (accessed on 1 March 2024), the Canadian Cancer Statistics Dashboard at https://cancerstats.ca/ (accessed on 9 May 2024), Canadian Partnership Against Cancer’s 2018 Cancer System Performance Report at https://s22457.pcdn.co/wp-content/uploads/2019/01/2018-Cancer-System-Performance-Report-EN.pdf (accessed on 14 March 2024), and Statistics Canada’s 2021 Census of Population Profile at https://www12.statcan.gc.ca/census-recensement/2021/dp-pd/prof/index.cfm?Lang=E (accessed on 14 March 2024).
